# Nitric oxide regulation of temperature acclimation: a molecular genetic perspective

**DOI:** 10.1093/jxb/erab049

**Published:** 2021-02-05

**Authors:** Inmaculada Sánchez-Vicente, Oscar Lorenzo

**Affiliations:** 1 Departamento de Botánica y Fisiología Vegetal, Instituto Hispano-Luso de Investigaciones Agrarias (CIALE), Facultad de Biología, Universidad de Salamanca, C/ Río Duero 12, 37185 Salamanca, Spain; 2 Université Paris-Sud, France

**Keywords:** Cold stress, gasotransmitter, GSNO reductase (GSNOR), heat stress, nitrate reductase (NR), reactive oxygen species (ROS), transcription factors (TFs)

**The current environmental situation is dominated by climate change, including heat and cold waves that trigger adverse conditions for plant growth and development. Identification of key signalling molecules during stress-related events can help in the development of strategies to mitigate detrimental effects and to improve plant tolerance. Among stress regulators, nitric oxide (NO) has emerged as a central gasotransmitter involved in the control of adaptive responses, acting to tailor plant growth and stress responses. Here, we outline the implications of this control by NO and highlight the genetic and molecular evidence for its role in enhancing tolerance and adaptation to heat and cold stress, which in turn has revealed possible new approaches for confronting future environmental challenges.**

## Involvement of nitric oxide during tolerance to heat stress

As a consequence of climate change, increased temperatures have become an important threat to the maintenance of agriculture worldwide, and regional and local warming is predicted to have an even greater impact on biological systems over the coming years. Increases above optimum temperatures for plant development lead to what is broadly termed heat stress (HS), where serious damage is caused to growth and developmental processes. Several reports have associated high temperatures with molecular and physiological modifications that compromise the correct status of cells (see Box 1).

Box 1. **Schematic representation of plant molecular and physiological modifications in response to heat stress**

Heat stress (HS) disturbs the redox balance and affects the coordination of organelles and the structure and functionality of macromolecules (i.e. lipid oxidation, DNA damage, protein destabilization and aggregation, and disruption of enzyme activity; reviewed by Ohama *et al.*, 2017). Plants have evolved various mechanisms that control the signalling pathways involved in the acquisition of tolerance, including antioxidant defences and the accumulation of osmoprotectants, HEAT SHOCK FACTORS (HSFs), and HEAT SHOCK PROTEINS (HSPs). The figure compiles the effects of NO together with the specific molecular processes that act to improve plant tolerance to HS (created with BioRender. com).

Exogenous NO confers thermotolerance by increasing the activity of enzymes that scavenge reactive oxygen species (ROS), including SUPEROXIDE DISMUTASE (SOD), CATALASE (CAT), and ASCORBATE PEROXIDASE (APX), as observed in reed calluses and wheat plants (Hasanuzzaman *et al.*, 2012). In addition, NO has been reported to act upstream of CALMODULIN 3 (CaM3) in Arabidopsis, inducing thermotolerance by enhancing the binding of HSFs to DNA and increasing the accumulation of HSPs (Xuan *et al.*, 2010; Wang *et al.*, 2014), whilst NO metabolism is modulated by CaM3, which binds to GSNO REDUCTASE (GSNOR) under HS (Zhang *et al.*, 2020). There is an influx of calcium (Ca^2+^) into the cell in response to HS, and intracellular Ca^2+^ and H_2_O_2_ are known to act upstream of NO in the response to HS (Peng *et al.*, 2019). Indeed, both molecules increase the survival rate of rice seedlings by promoting the expression of *HSP26* (Uchida *et al.*, 2002).

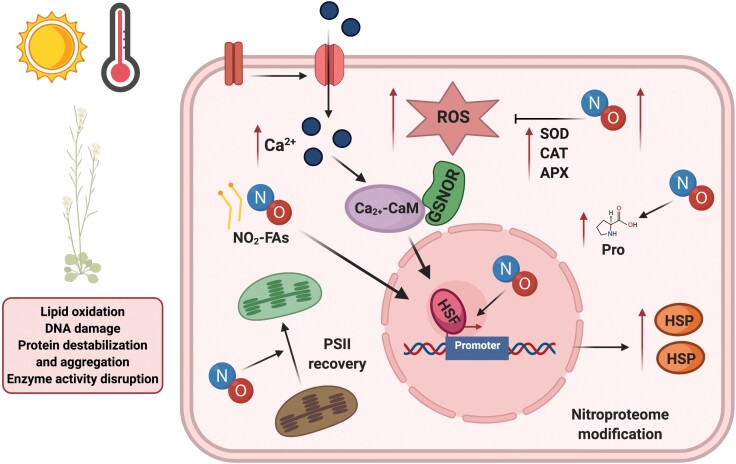

**Fig. 1.**
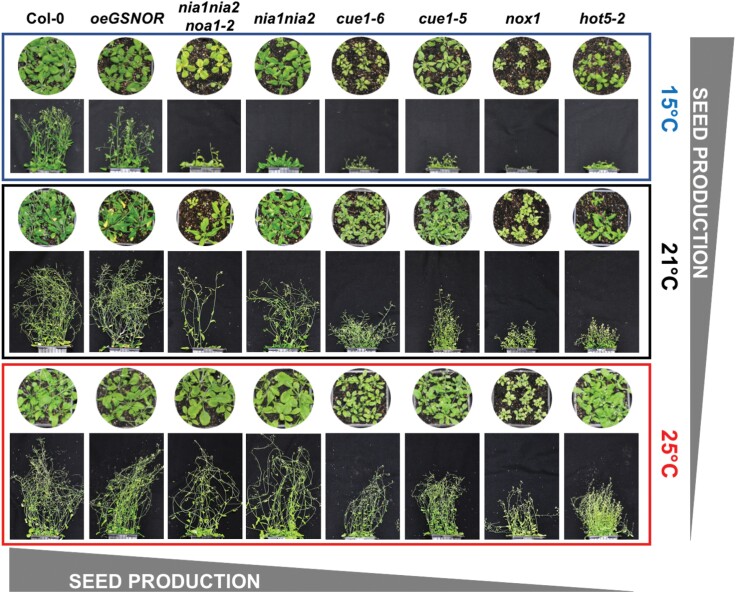
Phenotypes of nitric oxide (NO) homeostasis mutants and transgenic lines under the influence of different temperature regimes (±5 °C of the Arabidopsis normal growth optimum of 20–21 °C). The round images show plants at 4 weeks old and the rectangular images show plants at 6 weeks old. The Col-0 wild-type is shown together with the NO-deficient mutants *nia1 nia2*, which corresponds to impaired *NITRATE REDUCTASE* (*NIA/NR*) genes, and *nia1 nia2 noa1-2*, a triple-mutant defective in *NIA/NR* and *AtNOA1*-*NO-associated 1*; the NO-over-producer mutants *cue1-5* and *cue1-*6, related to the *chlorophyll a/b-binding [CAB] protein-underexpressed* gene, and *nox1-1*; the GSNO-over-producer mutant *hot5-2* (sensitive to hot temperatures); and a GSNO-deficient transgenic line overexpressing *GSNO* reductase (*oeGSNOR*). The genotypes are arranged according to the effects of temperature on total seed production.

Disturbed NO homeostasis deeply affects plant growth and development, for example as shown in Arabidopsis mutants either under- or over-producing NO, in S-nitrosoglutathione (GSNO) over-producer mutants, and in transgenic plants overexpressing GSNO reductase (Fig. 1). Although the plants grow faster with increasing temperature independently of the genotype (15<21<25 °C), the final seed production is compromised relative to the wild-type when NO and GSNO production is disturbed in both the NO- and GSNO-altered lines. These phenotypes suggest that maintenance of NO homeostasis is essential for adaptation to increasing temperatures and for a proper heat-stress response (HSR) to avoid severely reduced seed yields. Thus, the *hot5-2* and *nox1* mutants, which have altered levels of *S*-nitrosothiols (SNO) and NO, respectively, show thermotolerance defects that can be partially alleviated by the NO-scavenger 2-(4-carboxyphenyl)-4,4,5,5-tetramethylimidazoline-1-oxyl-3-oxide (cPTIO) (Lee *et al.*, 2008). In addition, mutants impaired in NO biosynthesis, such as *noa1* and *nia1 nia2*, exhibit lower rates of survival under HS conditions, which is reversible through exogenous treatments with NO donors (Xuan *et al.*, 2010). A large-scale analysis has also shown changes in the nitroproteome, revealing that tyrosine nitration is increased under HS (Chaki *et al.*, 2011).

NO initiates HSRs from multiple molecular features made up of a signalling network that includes activation of oxidative defences, accumulation of osmolytes and HEAT-SHOCK PROTEINS (HSPs), and protection of photosynthesis.

Heat shock promotes the production of reactive oxygen species (ROS) in plants (Wang *et al.*, 2014; Alamri *et al.*, 2019), which leads to cell dysfunction. To cope with this oxidative imbalance, plants readjust antioxidant systems to minimize cellular damage. NO accumulation has also been observed under elevated temperatures (Lee *et al.*, 2008) and counteracts this effect through the regulation of key enzymes that coordinate oxidative defence.

Osmotic adjustment protects cells against abiotic stresses, and plays a key role in the regulation of membrane fluidity, ROS scavenging, and protein stabilization. Among the solutes, proline (Pro) is widely considered to be a universal osmoprotectant that prevents damage to cells. Nevertheless, its role during HSR depends on the species, with Pro accumulation increasing the sensitivity of Arabidopsis and tobacco plants whilst conversely it might be crucial in providing better resistance in broad bean plants (Alamri *et al.*, 2019). Interestingly, the increase of NO during HS in broad bean correlates positively with Pro synthesis, which in turn improves plant thermotolerance.

Photosynthesis is a heat-sensitive process, and is mainly damaged by harmful effects in PSII. Exogenous NO ameliorates cell damage at the reaction centre and throughout the electron transport chain, improving PSII recovery (Chen *et al.*, 2013).

Among the crosstalk that occurs between NO and biomolecules, nitro-fatty acids (NO_2_-FAs) are considered as signalling molecules involved in HSR in animals, and nitro-linolenic acid (NO_2_-Ln) has been identified to play such a role in Arabidopsis, since exogenous treatments induce the expression of genes involved in HSR and the acquisition of thermotolerance, mainly HSPs and HEAT-SHOCK FACTORS (HSFs) (Mata-Pérez *et al.*, 2016).

Although a pivotal role for NO during HSR is clear, some studies have identified a reduction in the content of NO after exposure to HS (Chaki *et al.*, 2011). These apparently contradictory results might depend on the plant species, the tissue, and the specific heat treatment, as well as the highly dynamic nature of NO.

## Association of nitric oxide with cold stress

Similar to HS, low temperatures represent one of the most harmful abiotic stresses, resulting in significant damage to plants, and consequently to potentially extreme yield losses. Under cold stress, plants suffer changes in both biochemical and physiological processes, and as a result they have evolved several strategies to minimize the effects (see Box 2). Cold acclimation constitutes a highly complex process that can confer great resistance to exposure to low, non-freezing temperatures. Several NO genetic tools (e.g. Fig. 1) and NO-mediated mechanisms for coping with cold stress and improving tolerance have been described.

Box 2. **Schematic representation of plant molecular and physiological modifications in response to cold stress**

Cold stress provokes membrane damage, cellular dysfunction, an imbalance of metabolites, and disruption of enzyme activity. NO controls this cold-responsive network by scavenging reactive oxygen species (ROS) and by causing reductions in the levels of polyamines, anthocyanins, flavonoids, sugars, and phytohormones such as abscisic acid (ABA) and jasmonates (JA) (Costa-Broseta et al., 2018, 2019). The figure compiles the effects of NO together with the specific molecular processes that act to improve plant tolerance to cold stress (created with BioRender.com).

Exogenous NO treatment leads to a reduction in the level of malondialdehyde (MDA), whilst chlorophyll content is increased and SUPEROXIDE DISMUTASE (SOD) and ASCORBATE PEROXIDASE (APX) activities are promoted. In addition, it has been found that the source of NO production during the cold response originates mainly from the activity of NITRATE REDUCTASE (NR) (Zhao *et al.*, 2009; Cantrel *et al.*, 2011; Lv *et al.*, 2017; Sehrawat *et al.*, 2019). It has been reported that cold acclimation stimulates NR activity and transcription of *NITRATE REDUCTASE 1* (*NIA1*), thus increasing the NO content, a result that is supported by a lower degree of freeze tolerance being observed in the Arabidopsis *nia1 nia2* mutant (Zhao *et al.*, 2009). This suggests that the maintenance of correct NO levels is highly important.

Recent studies have examined this apparent crosstalk and have indicated that H_2_O_2_ is involved in the induction of NO by brassinosteroids (BRs) under cold stress (Arfan *et al.*, 2019). This NO accumulation has been found to be essential during the induction of alternative oxidases, which is dependent on BR signalling and ultimately contributes to the protection of the photosystem (Arfan *et al.*, 2019). Furthermore, NO is known to be a signal that acts downstream of H_2_O_2_ and cooperates with jasmonates during the acquisition of freeze tolerance (Liu *et al.*, 2019).

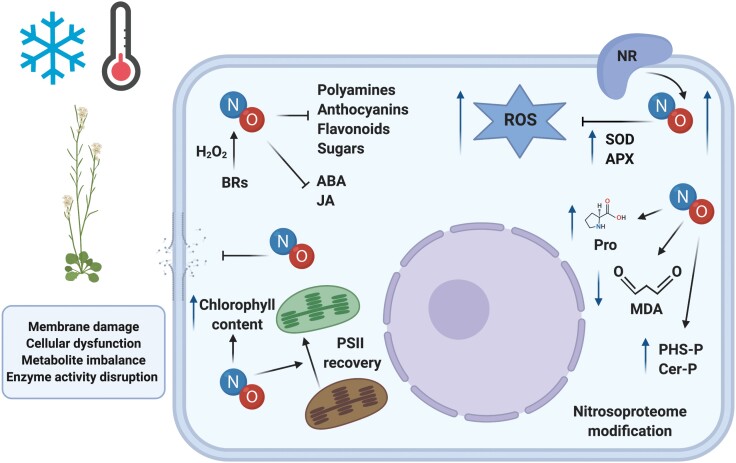

Large-scale analyses have shed light on the functions of NO during plant responses to low temperature, which involve changes in hormonal pathways, accumulation of osmoprotectants, and oxidative responses. NO acts to attenuate the freezing response, modulating the switch between constitutive feedback and normal growth under optimal conditions. This explains the cold-related phenotype observed in the *nia1 nia2 noa1-2* triple-mutant, which is characterized by increased survival under cold stress (Costa-Broseta *et al.*, 2018) but with a lower capacity to tolerate freezing conditions when compared to cold-acclimated wild-type plants (Costa-Broseta *et al.*, 2019). Nevertheless, earlier work reported a lower survival rate for the double-deficient mutant *nia1 nia2* (Zhao *et al.*, 2009). In a similar way to HS, these apparently contradictory observations might be explained by the different conditions used to carry out the experiments.

NO is able to modify the nitrosoproteome in *Brassica juncea* and Arabidopsis during the first hours of exposure to cold stress, highlighting its involvement in post-translational regulation. The most common mechanisms that are susceptible to modification by NO are those related to photosynthesis, redox homeostasis, metabolism, and signalling pathways. Although studies have shown that nitrosated proteins are clearly accumulated, only about 30% of those identified are consistent between the different analyses reported, which emphasizes the highly dynamic nature of NO regulation. Other recent research has also drawn attention to the subcellular changes that occur when NO is attached to cysteine residues, suggesting the existence of nitrosation-mediated nuclear trafficking that has an impact on cellular metabolism and redox status (Sehrawat *et al.*, 2019). These findings are also supported by results obtained in bermudagrass that show that NO has a crucial role in maintaining cell membrane stability, in antioxidant responses, and in PSII recovery, with temperature changes involving interconnections between H_2_O_2_ and NO during the plant responses (Fan *et al.*, 2015).

Similar to HS, Pro is linked to cold tolerance, and its levels are modulated by NO production via NITRATE REDUCTASE (NR), which stimulates its synthesis and reduces its degradation through the control of *P5C SYNTHASE1* (*P5CS1*) and *Pro DEHYDROGENASE* (*ProDH*), respectively (Zhao *et al.*, 2009).

Among the transduction molecules related to cold perception, lipid-derived signals represent one of the earliest responses. NO is involved in the formation of these signals, and it specifically regulates the production of phytosphingosine phosphate (PHS-P) and ceramide phosphate (Cer-P) (Cantrel *et al.*, 2011). Overall, studies consistently indicate that NO is crucial for providing increased resistance to the harmful effects of cold stress.

## Perspective

Exposure to extreme conditions outside of optimal temperatures reduces both crop yield and quality. NO has been shown to be a crucial gasotransmitter during temperature acclimation and as such it holds great promise for contributing to our assessments of how plants respond to climate change. Advances in research on temperature sensing have identified NO as a key temperature-sensitive mediator that helps to ensure that plants thrive under temperature-related stress. In order to improve and accurately focus future research, further knowledge is required regarding the molecular players that participate within the complex NO network, enabling us to differentiate the free NO and SNO contents, since their dynamics are not yet well understood. To this end, the plant material used, the developmental stages, and the specific treatments must all be carefully scrutinized as many contradictory results have arisen. To aid in this task, NO-controlled post-translational modifications (PTMs), such as *S*-nitrosation of cysteine residues, *S*-nitrosylation of metals, and nitration of tyrosine residues, constitute versatile signals that alter protein functionality through directly modifying stability, the ability to form multicomplexes, protein activity, and localization. The huge capacity for protein regulation influences adaptation to abiotic stresses. Protein 3D structural features should be analysed more in depth, since the proteins identified as putative NO targets through wide-scale analyses do not all present conserved primary sequences of adjacent amino acid residues for PTMs. The effects of NO_2_-FAs to directly drive *S*-nitrosation should also be considered. Furthermore, special attention should be paid to the potential use of the bZIP and NAC families of transcription factors as molecular players that respond to heat and cold stress, either as targets of PTMs or as regulators of NO homeostasis.
